# Case Report: Successful treatment of mismatch repair-deficient cervical esophageal adenocarcinoma with immune checkpoint inhibition

**DOI:** 10.3389/fonc.2026.1826912

**Published:** 2026-04-13

**Authors:** Aaron Dou, Chunzi Jenny Jin, Donna Maziak, Sebastien Gilbert, Jordan Sim, Eugene Leung, Moira Rushton

**Affiliations:** 1 Department of Medicine, University of Ottawa, Ottawa, ON, Canada; 2 Department of Radiation Oncology, University of Ottawa, Ottawa, ON, Canada; 3 Department of Thoracic Surgery, University of Ottawa, Ottawa, ON, Canada; 4 Department of Pathology and Laboratory Medicine, University of Ottawa, Ottawa, ON, Canada; 5 Department of Nuclear Medicine and Molecular Imaging, University of Ottawa, Ottawa, ON, Canada; 6 Department of Medical Oncology, University of Ottawa, Ottawa, ON, Canada

**Keywords:** case report, esophageal cancer, immunotherapy, microsatellite instability, mismatch repair deficiency, upper esophageal adenocarcinoma

## Abstract

Cervical (upper) esophageal adenocarcinoma is an extremely rare, aggressive, and biologically distinct subtype of esophageal cancer, with few cases reported in literature. As such, pathophysiology and treatment approaches remain poorly understood. We report a case of a 39-year-old male diagnosed with a locally advanced, unresectable, MMR-deficient cervical esophageal adenocarcinoma driven by double somatic mutation of the MLH1 gene. He was treated with pseudocurative intent treatment through combining standard of care (chemotherapy and immunotherapy) with high-dose radiation therapy. The patient achieved complete remission with no evidence of disease recurrence over three years after initial diagnosis. This case highlights the complex decision making that is involved in the treatment of rare cancers for which data from large randomized controlled trials are not available, and the importance of integrating trial data, tumor pathogenesis, biomarkers, and patient preferences.

## 1 Introduction

Adenocarcinoma of the cervical (upper) esophagus is extremely rare, and there is little consensus in the literature on diagnosis, treatment, and prognosis. In contrast, esophageal cancers overall are the 11th most prevalent cancer globally and the 7th leading cause of mortality amongst all malignancies (1). Five-year survival rates remain amongst the lowest of all cancers at 22%, alongside pancreatic (13%), liver (22%), and lung (27%) (2). The two main subtypes of esophageal cancer are squamous cell carcinoma and adenocarcinoma, with the latter primarily arising from the distal esophagus, consistent with its pathophysiological association with gastroesophageal reflux disease, Barrett esophagus, and obesity (3–5).

Pathophysiologically, upper esophageal adenocarcinomas (UEA) may arise from ectopic gastric mucosa or intestinal metaplasia, though specific driver molecular alterations are yet to be described (6–8). A 2013 case report by Verma et al. summarized existing literature regarding UEA, at which time there had been only 30 cases reported, with a mean age of diagnosis of 63 years and a male:female ratio of 9:1 (9). Surgery and radiation were the primary treatment modalities utilized.

In the past decade, additional studies have reported cases of UEA, corroborating its associations with ectopic gastric mucosa (10–18). Nevertheless, there remains a paucity of data surrounding treatment approaches for locally-advanced UEA as well as long-term outcomes. Regimens differed between induction *vs* adjuvant approaches; common regimens were generally based upon a fluoropyrimidine, platinum, and/or taxane agent, extrapolating data from trials for lower esophageal cancers. Additionally, to our knowledge, there are no reported cases of immunotherapy use for UEA treatment, even in light of recent trials in lower esophageal/gastro-esophageal junction (GEJ) cancers emerging such as KEYNOTE-975 and CHECKMATE-649 (19, 20).

We report a case of a patient with MMR-deficient (dMMR) cervical esophageal adenocarcinoma driven by double somatic loss of the MLH1 gene, who was treated with definitive chemoradiotherapy alongside immunotherapy with no evidence of disease recurrence at 3 years. We critically analyze the clinical decisions made, contextualizing this patient’s care within the overall landscape of UEA treatment.

Informed written consent was obtained from the patient for this study per institutional Research Ethics Board guidelines, and the patient reviewed the article prior to submission.

## 2 Case description

**Figure 1:**
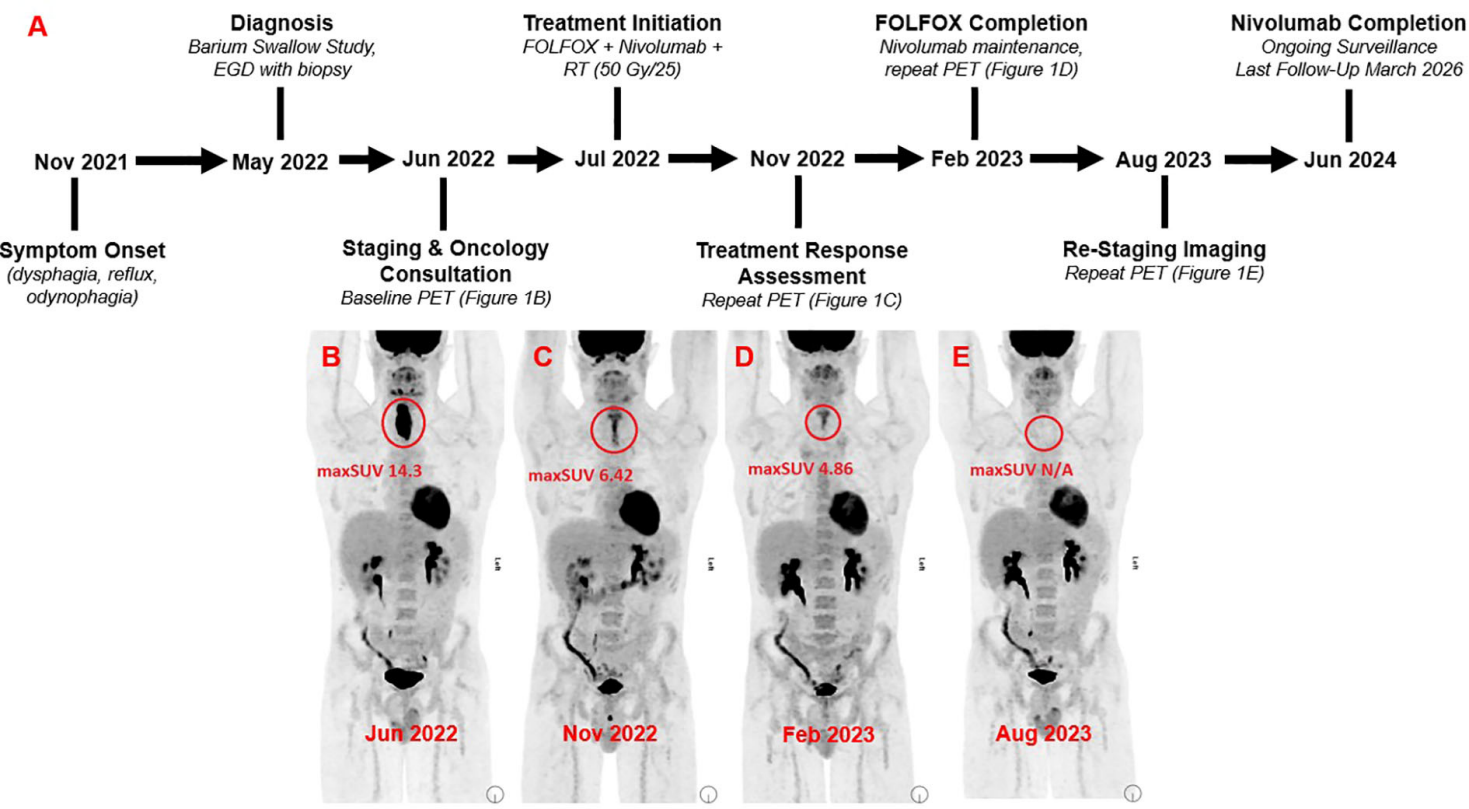
Timeline of patient presentation, diagnosis, treatment, and surveillance is shown in **(A)** The maximum standardized uptake value (maxSUV) of the primary tumour across serial positron emission tomography (PET) scans is illustrated in **(B)** (initial staging scan, June 2022, maxSUV 14.3), **(C)** (November 2022, maxSUV 6.42), **(D)** (February 2023, maxSUV 4.86), and **(E)** (no SUV uptake, August 2023).

In accordance with CARE guidelines, a case timeline is presented in **Figure 1A**.

### 2.1 Diagnostic assessment

A 39-year-old male with no known past medical history presented with a six-month history of progressive solid and liquid food dysphagia, odynophagia, and reflux, as well as a 20-pound unintentional weight loss over two months. He had neither smoking history nor second-hand smoke exposure and only consumed two cans of beer weekly. Family history was positive for two maternal aunts with colorectal cancer, and otherwise no known malignancy history.

**Figure 2:**
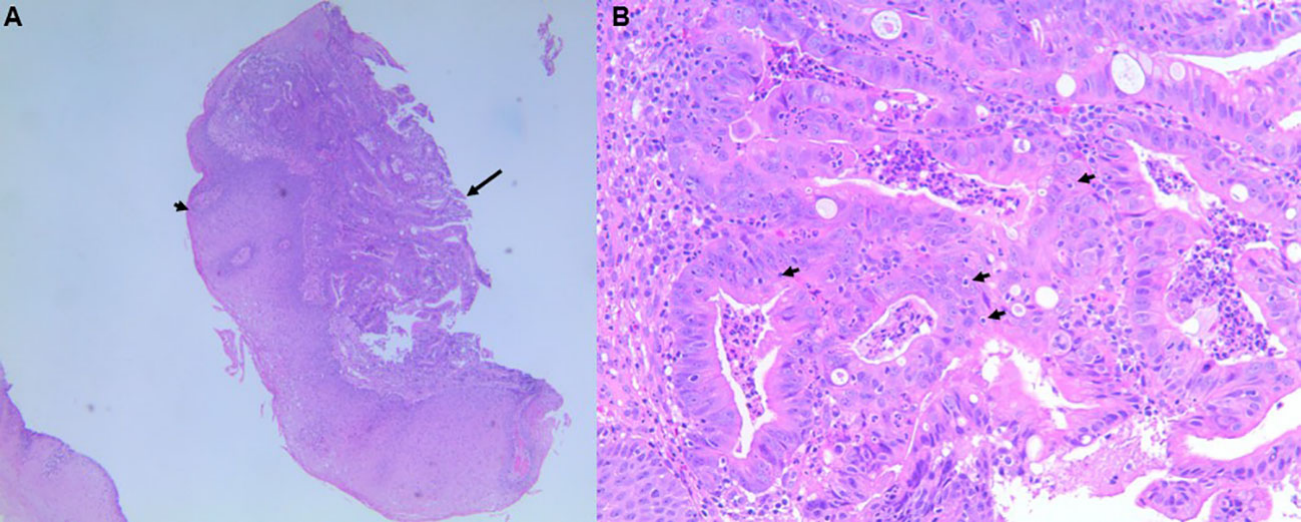
The pre-therapy biopsy, low power **(A)**, 40x magnification) and high power **(B)** 200x magnification) H&E images. The low power image in **(A)** shows the complex glandular proliferation of the adenocarcinoma (black arrow) undermining the squamous mucosa (black arrowhead) of the upper esophagus. The high magnification image in **(B)** shows the gland’s malignant features, including irregular nuclei and glandular complexity, in addition to intraepithelial lymphocytes (black arrowheads), which in some sites is a feature of the high neoantigen burden mismatch repair deficient carcinomas.

**Figure 3:**
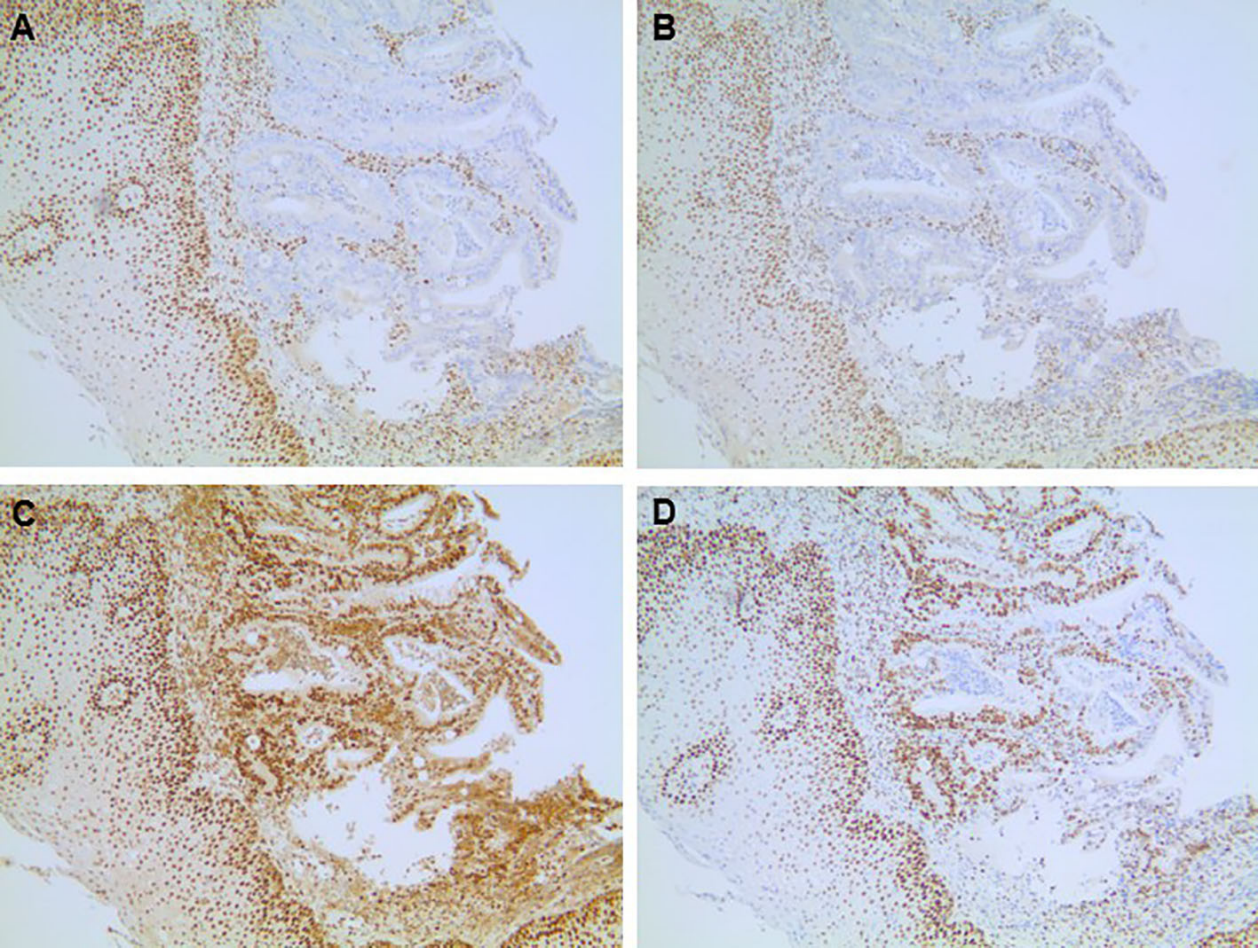
Mismatch repair protein immunohistochemical assays. MLH1 **(A)** 100x magnification) and PMS2 **(B)** 100x magnification) are lost in the malignant nuclei but retained in the surrounding squamous epithelium and lymphocytes. MSH2 **(C)** 100x magnification) and MSH6 **(D)** 100x magnification) are retained.

A barium swallow study was ordered which revealed an ulcerating cervical esophageal mass, and a subsequent esophagogastroduodenoscopy (EGD) demonstrated a 4cm obstructing lesion in the proximal esophagus between 18-22cm. Biopsies demonstrated invasive adenocarcinoma, at least intramucosal, that was moderately differentiated. HER2 was negative (0+) by immunohistochemistry (IHC), and tumor cells demonstrated loss of MLH1 and PMS2 expression with no MLH1 promoter hypermethylation. Pre-treatment pathology slides are shown in **Figures 2**, **3**. Initial staging CT of neck, chest, abdomen, and pelvis demonstrated a prominent 0.8cm right superior paratracheal node, which correlated with increased SUV intake on 18-FDG PET CT (**Figure 1B**). There were no distant metastases.

Given the pattern of expression of MMR pathway proteins (defect in the MutLα complex with loss of MLH1 and likely secondary PMS2 loss, with absence of MLH1 promoter hypermethylation) alongside family history of colorectal cancer, there was initial suspicion for Lynch syndrome (21). He was referred to genetics for further assessment. Blood testing revealed no germline pathogenic variants identified on a standard 31-gene gene panel associated with inherited cancer predisposition. Tumor testing was positive for double somatic MLH1 mutation. As such, no further genetic testing was pursued or recommended to his family.

### 2.2 Therapeutic intervention

He was referred to medical, radiation, and surgical oncology for further assessment and review. He was discussed at multi-disciplinary tumor board, and was determined to not be a candidate for surgical resection based upon the anatomic location of the tumor. Different options for systemic therapy were reviewed within the foregut medical oncology group:

Option 1: Cisplatin (75mg/m^2^) and 5-fluorouracil (4000mg/m^2^ over 4 days) given every 28 days for a total of 4 cycles, with concurrent radiation alongside the first 2 cycles. The total radiation dose would be 50 Gy in accordance with the INT 0123 trial, which demonstrated no statistical difference in survival between low-dose (50.4 Gy) and high-dose (64.8 Gy) radiation (22). This approach is consistent with regimens used by the previously described UEA case studies.

Option 2: Palliative-intent chemotherapy and immunotherapy per the CHECKMATE-649 study, which compared nivolumab plus chemotherapy versus chemotherapy alone in the treatment of unresectable gastric, GEJ, and esophageal adenocarcinomas (20).

Option 3: Combining standard of care (chemotherapy and immunotherapy) with radiation therapy as part of pseudocurative intent treatment, despite the patient’s tumor being unresectable due to location, considering his young age and excellent functional status. We discussed enrollment in clinical trials, namely KEYNOTE-975, which was ongoing at time of diagnosis. This study investigated pembrolizumab *vs*. placebo in conjunction with definitive chemoradiotherapy as first-line treatment for patients with locally-advanced, unresectable esophageal and GEJ tumors (19). Ultimately, he was not eligible for enrollment as trial guidelines stipulated that all future patients at our center enrolled in KEYNOTE-975 receive 60 Gy of radiation; however, our site had already committed to a dose of 50 Gy for patients with lower esophageal cancers.

**Figure 4:**
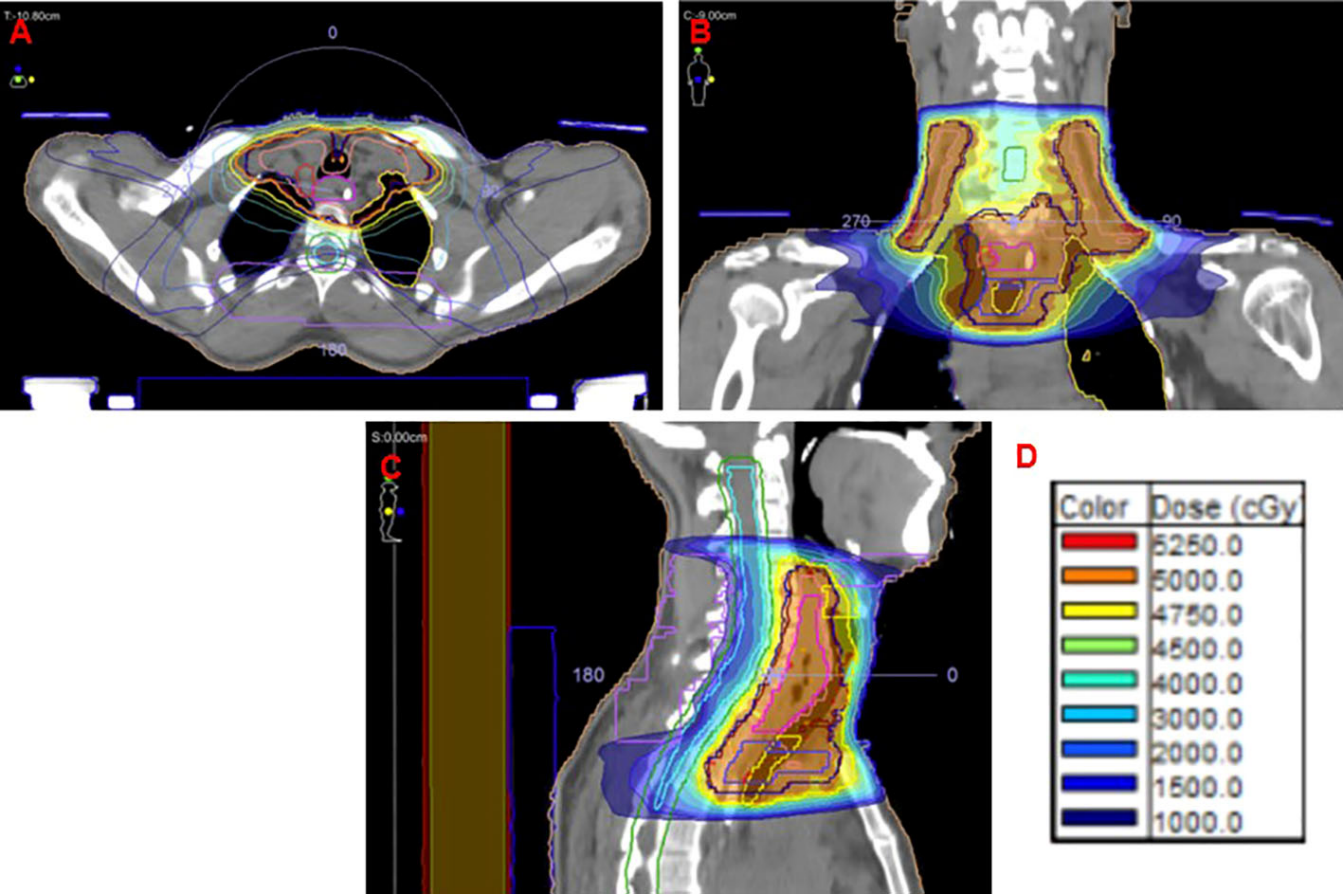
Radiation therapy treatment plan. **(A)** Axial, **(B)** coronal, nd **(C)** sagittal views from the treatment planning CT showing target volumes isodose distributions. **(D)** Color scale depicting radiation dose levels in centigray (cGy) corresponding to Panels **(A-C)**.

Integrating the evidence above and the patient’s preferences and goals of care, we pursued pseudocurative, “radical” treatment combining FOLFOX and nivolumab per CHECKMATE-649 with radiotherapy. Radiation was prescribed as 50 Gy in 25 fractions using a standard conformal arc (VMAT) technique. The radiation treatment plan and target volumes are displayed in **Figure 4**. He was planned to receive 12 cycles of FOLFOX + Nivolumab, followed by maintenance nivolumab alone for as long as tolerated and effective, or up to 2 years.

The patient initially tolerated treatment well with grade 1 fatigue and neuropathy. However, prior to cycle 4, due to effects of cumulative toxicity from radiation and systemic therapy, he experienced worsening grade 3 dysphagia and esophagitis. The dose of chemotherapy was reduced thereafter to 80% ideal dose, and chemotherapy was omitted for a total of 2 cycles (cycles 4 and 8). EGD was performed to work-up the cause of his symptoms, revealing an esophageal stricture, likely secondary to his cancer and radiotherapy, which was dilated with good response.

A repeat PET scan was performed prior to cycle 10 of treatment to assess treatment response, which showed significant reduction in the size and FDG avidity in the esophageal mass, with no other areas of uptake (**Figure 1C**). To account for omission of chemotherapy during C4 and C8, his treatment plan was adjusted to maintain a total of 12 cycles of chemotherapy. By the final few cycles of his treatment, the patient’s fatigue, dysphagia, odynophagia, and solid food intake had significantly improved, corresponding with a 5-pound weight increase. He successfully completed 12 total cycles of FOLFOX alongside nivolumab.

### 2.3 Follow-up and outcomes

The patient commenced maintenance nivolumab in February 2023. He received a re-staging esophageal biopsy at this time, which was negative for residual disease, alongside PET which showed moderate SUV uptake in the area of malignancy (4.86, down from maximum 6.42 previously, as shown in **Figure 1D**). His only symptom was mild ongoing dysphagia secondary to radiation-induced stricture, necessitating a repeat esophageal dilation around 6 months following completion of chemotherapy with improvement in symptoms. At this time, a final repeat PET scan was performed which showed zero residual SUV uptake (**Figure 1E**).

He continued maintenance nivolumab for a total of 2 years, which he tolerated well without any adverse effects related to treatment. From a surveillance standpoint, he received CT scans of the chest, abdomen, and pelvis every 3 months, which showed no signs of distant disease or local recurrence. Serial EGD was not pursued given improvement in his dysphagia post-esophageal dilation, though would have been considered if he had symptomatic recurrence. Three years after initial diagnosis, the frequency of follow-up appointments and surveillance imaging was reduced to every 6 months.

He continues to remain well symptomatically and psychologically, with no evidence of disease recurrence at most recent follow-up appointment in March 2026, nearly 4 years (46 months) after initial diagnosis. Ongoing follow-up is planned every 6 months for a minimum of 5 years post-initial diagnosis, with further follow-up schedule to be determined by informed discussion between the patient and medical oncology team.

## 3 Discussion

We report a case of a 39-year-old male diagnosed with moderately differentiated cervical esophageal adenocarcinoma, who was successfully treated with FOLFOX and nivolumab per CHECKMATE-649 protocol alongside concurrent radiation as radical treatment for unresectable disease. Initial molecular markers were concerning for Lynch syndrome though subsequent genetic testing revealed double somatic loss of MLH1. He tolerated treatment well with minimal long-term toxicity. He is disease-free nearly 4 years post-initial diagnosis.

The treatment of this patient highlights several important principles both in the treatment of UEA, as well as more generally in the management of rare cancers for which guidelines and high-quality evidence are unavailable. Our patient’s treatment plan was guided by interpretation of previous trial data in the context of their tumor’s molecular characteristics. A subgroup analysis of CHECKMATE-649 amongst patients with microsatellite instability-high (MSI-H)/dMMR tumors demonstrated large benefit for overall survival (OS) for FOLFOX + nivolumab compared to chemotherapy alone (HR = 0.33) (20). The high sensitivity of MSI-H tumors to immune checkpoint inhibitors (ICI) has been corroborated by several other trials in GI cancer populations, including KEYNOTE-177, KEYNOTE-158, and CHECKMATE-142 (23–27). This has led to tumor-agnostic, histology-independent approval of pembrolizumab for MSI-H or dMMR tumors (28). Our patient’s robust and sustained response aligns with this data. Future basket trials may further highlight the role of ICI for specific molecular alterations.

The presence of double somatic mutations in MLH1 is not uncommon in dMMR tumors. Several studies have highlighted that in patients with dMMR tumors without MLH1 promoter hypermethylation (termed as having “suspected Lynch syndrome”), over 50% have somatic biallelic loss of MMR proteins (29–31). As a corollary, all patients with suspected Lynch syndrome should have both somatic and germline testing as this may guide future genetic testing for the patient and their family.

The finding of MMR deficiency in our patient’s tumor is also interesting from a mechanistic standpoint for UEA pathogenesis. Of the case reports we reviewed from the past decade, only two commented upon MMR status: one tumor was MMR proficient, the other occurred in a patient with MSH-2 related Lynch syndrome arising from a cervical gastric inlet patch (15, 18). This raises the hypothesis that MMR deficiency could be a possible contributing factor in the pathogenesis of UEA arising from heterotopic gastric mucosa. Recent large analyzes and EORTC position statements highlight MMR deficiency as relatively rare in esophageal adenocarcinomas overall (32, 33). However, as described above, UEAs may be biologically and molecularly distinct. One study of 75 patients with gastric intestinal metaplasia found that MSI can emerge even prior to carcinogenesis, suggesting it could play a role in the metaplasia-dysplasia-carcinoma sequence (34). Several of the case reports on UEA have also commented on an intestinal metaplasia phenotype, lending biologic plausibility to this hypothesis, though caution must be taken in not overinterpreting the role of MMR (which could function as a passenger biomarker) without more high-quality data (13, 35). This would be an interesting area for future exploration.

Our study also highlights the importance of considering patient preferences, frailty, and overall health status alongside tumor-related factors when making treatment decisions. In this case, our patient’s young age and absence of significant comorbidities supported the use of chemotherapy and immunotherapy alongside radiotherapy. Furthermore, through discussion of goals of care, he was highly motivated to pursue any combination of treatment that would most reduce risk of recurrence despite possibility of increased treatment toxicity. In other patients with increased frailty, this treatment approach may have higher risk for serious treatment-related adverse events or otherwise confer toxicity that may be unacceptable for a patient’s quality of life. In these cases, other treatment options highlighted above may be considered, including palliative-intent chemotherapy and immunotherapy or chemotherapy and radiotherapy without immunotherapy.

In summary, our study adds to the growing body of case reports of UEA, interpreting and summarizing existing literature while critically analyzing treatment decisions. To our knowledge, this is the first reported case of successful treatment of UEA with ICI with sustained remission over 46 months. We highlight the importance of understanding tumor pathogenesis and biomarkers in the selection of treatment for rare cancers and postulate future directions for mechanistic exploration of UEA. As a reminder of the human impact of our work in oncology, and consistent with CARE guidelines (36), we conclude with a statement from our patient himself regarding his cancer journey.

## 4 Patient perspective

“You have cancer” was the last thing I thought I would hear. As an otherwise healthy, non-smoker, non-drinker, who was active and ate well, this possibility never even crossed my mind. I started having throat pain and discomfort in November of 2021. I attributed it to lingering effects of a bad cold or Covid. Then, I started having difficulty swallowing. My family doctor referred me for a swallowing study on May 20, 2022 at 11am, the Friday before a long weekend. My doctor called me the very next day. He said there was no easy way to tell me this, but I had a large tumor in my esophagus. I was in shock and in disbelief, but knew that I had a fight ahead of me. How hard a fight it would be, I would only come to understand in the weeks and months to come.

I received an urgent biopsy. The results came back quickly, and it was as feared, active cancer. The details about everything are fuzzy, but I do remember that the severity of my situation was laid out in front of me as per my request, 30% of patients survive what I had. I made it clear to my oncologists that I would do whatever I needed to do. We made the decision to pursue aggressive chemo, high level radiation, as well as immunotherapy. I knew that I’d have to do my part and fight with everything I had. I truly had no idea how hard this would be. The chemo made me feel weak and sick, and after finishing the radiation, I had severe burns on my neck and in my throat. Through discussion with my oncologist, we decided to pause chemo for 1 round to let me heal. I was worried I was disappointing her, but she assured me that I was a warrior and was not doing anything that wasn’t common. Most people do not go through their treatment schedule without a hiccup or two.

In the months to follow, I started to heal. It was a long slow road to eating normally again and gaining back some weight it’d lost. I continued with immunotherapy for a further 18 months or so. The question I had for my doctor was, “did we accomplish what you had hoped we would”? Throughout the journey, tests, and PET scans, we had constantly gained traction, and the cancer had not metastasized. This gave me incredible strength to keep pushing despite the nasty side effects and pain. I probably shouldn’t have made it, but so many things came together to give me a chance of survival. Had the radiation and chemo drugs not been given, then I might not be alive to write this. I truly feel that the addition of immunotherapy was pivotal in beating the cancer. The 3 treatments put together, the incredible team that listened and monitored me so closely and pushed so hard to get me into treatment ASAP, and my will to fight and not quit no matter what: all this got me to the other side.

## Data Availability

The original contributions presented in the study are included in the article/supplementary material. Further inquiries can be directed to the corresponding author.
